# Outcomes and treatment responses, including work productivity, among people with axial spondyloarthritis living in urban and rural areas: a mixed-methods study within a national register

**DOI:** 10.1136/annrheumdis-2020-216988

**Published:** 2020-06-10

**Authors:** Rosemary J Hollick, Kevin Stelfox, Linda E Dean, Joanna Shim, Karen Walker-Bone, Gary J Macfarlane

**Affiliations:** 1 Aberdeen Centre for Arthritis and Musculoskeletal Health, Epidemiology Group, University of Aberdeen, Aberdeen, UK; 2 MRC Versus Arthritis Centre for Musculoskeletal Health and Work, University of Aberdeen, Aberdeen, UK; 3 Epidemiology Group, School of Medicine, Medical Sciences and Nutrition, University of Aberdeen, Aberdeen, UK; 4 MRC Versus Arthritis Centre for Musculoskeletal Health and Work, University of Southampton, Southampton, UK

**Keywords:** epidemiology, qualitative research, spondyloarthritis, outcomes research

## Abstract

**Objective:**

To examine differences in clinical and patient-reported outcomes, including work, in individuals with axial spondyloarthritis (axSpA) living in rural and urban settings.

**Methods:**

Using a sequential, explanatory mixed-method design, data from the British Society for Rheumatology Biologics Register for Ankylosing Spondylitis were used to (1) characterise participants with axSpA living in rural and urban areas and (b) assess any differences in outcome after commencement of biologic therapy (phase 1). Semistructured interviews (phase 2) further explored the results from phase 1.

**Results:**

Patients with axSpA living in rural areas were older and more likely to work in a physical job. Among patients prescribed biologics, there were no differences in response to biologics, but after adjustment for age, sex and local area deprivation rural dwellers reported more presenteeism and overall work impairment. Work effects could be explained by accounting for individual differences in disease activity, fatigue, physical function and job type. Interviews highlighted the complex relationship between clinical factors, contextual factors (work environment, job demands) and work disability. The ability to work and flexibility in terms of what, when and how tasks are undertaken were important. Support from employers was variable and healthcare professionals were often perceived as unsupportive.

**Conclusions:**

Patients with axSpA living in rural areas report a greater impact of their disease on work productivity. New measures are needed to capture important contextual factors and comprehensively determine the impact of long-term conditions on work. Future European League Against Rheumatism axSpA recommendations should include support to work as a target to optimise quality of life in patients with axSpA.

**Key messages**

**What is already known about this subject?**

- Work productivity is significantly affected by axial spondyloarthritis (axSpA).

**What does this study add?**

- The ability to work is of prime importance to patients with axSpA.
- This is the first paper to demonstrate that individuals with axSpA living in rural areas report a greater impact of their disease on their ability to work.
- This mixed-methods study highlights the relevance and nature of clinical and contextual factors, and offers new insights into how features of occupations, individuals and workplaces influence work disability in axSpA.

**How might this impact on clinical practice or future developments?**

- Impaired ability to work for those living in rural settings has important consequences as the ageing, multimorbid population is increasing faster in rural areas and people are working to older ages.
- New comprehensive work measures are needed to capture important contextual factors and the impact of long-term conditions in the workplace.
- Future European League Against Rheumatism axSpA recommendations should include workability as a target for management to optimise quality of life in patients with axSpA.

## Introduction

The ability to participate in the labour market is not only important economically, but work also has a positive impact on social and psychological health.^1^ While absenteeism and job loss are the endpoints of work disability, these are only the tip of the iceberg because musculoskeletal disorders have a substantially greater impact on worker productivity.^2^

According to one estimate, work disability affects up to 30% of patients with axial spondyloarthritis (axSpA).^3^ Compared with the general population, individuals with axSpA are three times more likely to withdraw from work, especially from physically demanding jobs.^4^ Other factors associated with work disability in axSpA include older age, longer disease duration, functional impairment, pain, fatigue, low mood, comorbidity and lower educational attainment.^5 6^ We have previously shown that job type and disease activity predict presenteeism (being in the workplace but performing below their usual levels (productivity loss) because of ill health), which in turn predicts absenteeism and job loss.^7^ While biologic therapy reduces presenteeism and improves functional capacity in axSpA, significant unmet need remains in terms of work disability.^8^

There is good evidence that supportive work environments are important to enable individuals with health conditions to work; however, not all workplaces are created equally, and in particular there are important rural–urban differences.^9^ While employment rates may be higher, incomes tend to be lower in rural areas, with restricted choice and opportunities for career advancement.^10^ Rural workers more often work in smaller businesses and industries, and manual, lower paid jobs are more prevalent. Such jobs may be less flexible and have more restrictions preventing adjustments (eg, paid leave) than higher-income jobs.

With the ageing, multimorbid population increasing faster in rural areas,^11 12^ and people working to older ages, understanding the relationship between rurality, health and work outcomes is important to ensure that individuals with rheumatic disease are supported to live and work well. A number of studies have highlighted inadequate access to specialist healthcare services for those with rheumatic and musculoskeletal disease (RMD) living in rural and remote locations.^13–16^ However, in a recent systematic review of the literature, we found very limited evidence as to whether outcomes are poorer for individuals with rheumatic disease living in rural settings.^17^ No eligible studies examined work outcomes. All studies were relatively small, rarely provided a definition of rural status, and did not measure aspects related to rurality, such as access to services, patient preferences or the work environment. Furthermore, important differences in socioeconomic status between rural and urban areas were noted but not usually adjusted for when comparing outcomes, making it difficult to determine whether rurality is an independent predictor of poor outcome in RMD or a surrogate marker for socioeconomic status.^17^

This study aims to examine differences in clinical and patient-reported outcomes, including work outcomes, in individuals with axSpA living in rural and urban settings, using a sequential, explanatory mixed-method design.^18^ First, data from the British Society for Rheumatology Biologics Register for Ankylosing Spondylitis (BSRBR-AS) will be used to (1) describe the characteristics of participants with axSpA living in rural and urban areas and (2) assess any differences in outcome after commencement of biologic therapy between these groups (phase 1). This will be followed by semistructured interviews in a subset of registry participants (phase 2) to further explore the results from phase 1.

## Materials and methods

### Phase 1: BSRBR-AS data

The BSRBR-AS is a prospective cohort study which recruited biologic-naïve adult patients meeting the Assessment of SpondyloArthritiS (ASAS) criteria for axSpA, from 83 centres across Great Britain in 2012–2017.^19^ The full study protocol has been previously published.^20^ This analysis uses clinical and patient-reported data from recruitment (baseline) for all subjects and prospective data from the subcohort commencing biologic therapy at baseline. Their ‘outcome’ is measured at the first time point between 6 weeks and 12 months after starting therapy. Clinical data were collected during rheumatology appointments and patient-reported outcomes via postal questionnaires.

#### Clinical data

The following were systematically collected: age, gender, prevalent extraspinal manifestations, which medical imaging was undertaken (X-ray and MRI), medication (non-steroidal anti-inflammatory drugs (NSAIDS) and disease-modifying antirheumatic drugs (DMARDS) use in previous 6 months), human leukocyte antigen B27 (HLA-B27) status, and Bath Ankylosing Spondylitis Metrology Index score (scored from 0 (least) to 10 (most severe)).^21^ We calculated the lag time from the year of symptom onset to the date of first rheumatology assessment.

#### Patient-reported measures

The questionnaires enquired about educational level, smoking status, alcohol intake, employment status, and among those employed the job type (mainly desk/sedentary or mainly physical/labour-intense). The Work Productivity and Activity Impairment Specific Health Problem indicated the proportion of work missed (absenteeism) or impaired (presenteeism) over the previous week, measure of overall work impairment (absenteeism and presenteeism) and other activity impairment outside of work (all scored 0%–100%).^22 23^ Quality of life was assessed by the Ankylosing Spondylitis Quality of Life Index (scored from 0 (best) to 18 (worst)^24^) and the Short Form-12 Physical and Mental Component Scores (scored from 0 (worst) to 100 (best)^25^). The Hospital Anxiety and Depression Scale (HADS) indicated mental health (scored from 0 (best) to 21 (worst)^26^), while fatigue and sleep disturbance were measured using the Chalder Fatigue Scale (from 0 (best) to 11 (worst)) and Jenkins Sleep Evaluation Questionnaire (from 0 (best) to 20 (worst)), respectively.^27 28^ Spinal pain was assessed using a 10 cm Visual Analogue Scale and the Bath Ankylosing Spondylitis indices used to measure disease activity (BASDAI), physical function (BASFI) and patient global score (BASG) (all scored from 0 (best) to 10 (worst)).^29–31^ Postcodes, provided by participants on returned questionnaires, were used to:

- Derive a measure of local area deprivation (from 1 (most deprived) to 5 (least deprived))^32 33^ in order to adjust for local-level deprivation.
- Categorise participants into those living in rural and urban areas by the 2011 UK Census.^32 33^ Rural areas were categorised as settlements of less than 3000 people.

### Phase 2: semistructured interviews

Semistructured telephone interviews were conducted with a subset of BSRBR-AS registry participants from phase 1 who had consented to participate in further studies. The focus and content of the interviews were determined by the results of phase 1. As differences in presenteeism emerged as an important factor, the interview topic guide (see online supplementary text) was developed to explore this further, informed by the findings from phase 1 and review of existing qualitative literature on work outcomes in axSpA and other rheumatic diseases, as well as input from patient partners. BSRBR-AS participants were purposively selected for interview based on employment status, job type, age, disease duration and urban or rural location across seven geographical sites within the UK. Interviews explored interactions between aspects of disease (such as activity, function, fatigue, medications), job characteristics and work environment, and their impact on work. We also examined the impact of work disability on quality of life, career choice and progress and support to work. All interviews were conducted by a single researcher (KS) previously unknown to study participants.

10.1136/annrheumdis-2020-216988.supp1 Supplementary data

### Data analysis

The characteristics of patients living in urban and rural areas were described in terms of mean (and SD) for continuous variables and proportions for categorical variables, with differences assessed via Mann-Whitney and χ^2^ tests. Relationships between living in a rural area and work outcome (absenteeism, presenteeism and overall work impairment) were further quantified using linear regression models, adjusted for age, gender, local area deprivation and baseline scores (for patient-reported measures). Sequential adjustments were then made for additional factors previously found associated with presenteeism (disease activity (BASDAI), fatigue, job type and physical function (BASFI)).^34^ Results are given as β coefficients and 95% CI. Among those commencing a biologic agent, the potential relationship between rural living and outcome at 12-month follow-up was assessed. Outcome was evaluated in two main ways:

- Meeting response, as defined by the ASAS improvement criteria (ASAS20 and ASAS40).^35 36^
- Patient-reported measures (adjusted for baseline score): disease activity, physical function, quality of life, fatigue, sleep disturbance, anxiety, depression, activity impairment and work outcomes (presenteeism, absenteeism and overall work impairment).

Analyses were conducted on STATA V.15.0 using the August 2017 version of the BSRBR-AS data set.

Interviews were audio-recorded, transcribed verbatim and entered into NVivo V.12 software to facilitate data organisation and retrieval. Interviews were analysed thematically by KS, supported by RJH, using a combination of anticipated themes (informed by the topic guide) and inductively derived coding.^37^ The analytical process involved (1) familiarisation with data and initial coding; (2) organisation of codes according to similarity of meaning; (3) development and review of (sub)themes; and (4) definition of (sub)themes and their interactions. Emerging analysis was discussed and developed with all authors. Data saturation was deemed to have been achieved through thematic and code saturation.^38^

### Integration of data

Quantitative and qualitative data were integrated at several stages; analysis of the BSRBR-AS data in phase 1 was used to identify interview participants and to determine the focus and content of interviews in phase 2. Integration of findings from phase 1 and 2 provided a deeper understanding of the impact of axSpA on work and role of contextual factors.

### Patient and public involvement

Patient representatives on the BSRBR-AS strategy group identified work and the impact of geography on health outcomes as a key research priority. We developed this study to explore this. The interview topic guide was developed with input from patient partners. At the end of the study, the BSRBR-AS strategy group commented on findings and contributed to the dissemination plan.

## Results

### BSRBR-AS registry

#### Baseline characteristics of study population

There were 2390 BSRBR-AS participants eligible for the current study, of which 24% (n=579) lived in a rural area. Rural dwellers were older (50.5 vs 47.4 years), less likely to be current smokers (15% vs 21%) and more likely to be in a predominantly physical/labour-intense occupation (54% vs 44%) (table 1).

**Table 1:** Baseline characteristics of urban and rural dwellers

		Urban dwellers		Rural dwellers		Difference (rural vs urban)
**Demographic characteristics**		**n**	**Mean (SD**)	**n**	**Mean (SD**)	**Mean difference (95% CI**)
Age*	Years	1810	47.4 (14.5)	579	50.5 (13.9)	3.1 (1.7 to 4.4)
Local area deprivation*	1 (most) – 5 (least)	1811	3.0 (1.4)	579	3.8 (1.0)	0.8 (0.7 to 0.9)
		**n**	**%**	**n**	**%**	**Mean % difference (95% CI**)
Gender	Male	1242	68.6	394	68.0	−0.6 (−8.3 to 7.1)
Smoking status*	Never	597	43.8	206	45.2	1.4 (−5.6 to 8.4)
	Ex	475	34.8	184	40.3	5.5 (−1.0 to 12.0)
	Current	291	21.3	66	14.5	−6.8 (−10.9 to −2.7)
Alcohol	Never	94	6.9	30	6.6	−0.3 (−2.8 to 2.2)
	Ex	243	17.9	78	17.1	−0.8 (−5.1 to 3.5)
	Current	1021	75.2	348	76.3	1.1 (−8.1 to 10.3)
Employed	Yes	528	38.4	175	37.9	−0.5 (−6.9 to 5.9)
Job type*	Mainly desk/sedentary	465	56.4	127	45.8	−10.6 (−20.0 to −1.2)
	Mainly physical/labour-intense	360	43.6	150	54.2	10.6 (0.9 to 20.3)
Clinical characteristics						
HLA-B27 status*	Tested	1203	66.4	412	71.2	4.8 (−3.0 to 12.6)
NSAID	Prescribed (last 6 months)	1340	74.6	431	75.0	0.4 (−7.7 to 8.5)
DMARD	Prescribed (last 6 months)	170	13.4	63	15.7	2.3 (−1.9 to 6.5)
Images taken	X-ray	1493	82.4	490	84.6	2.2 (−6.3 to 10.7)
	MRI*	1225	67.6	361	62.3	−5.3 (−12.7 to 2.1)
ESM (history)	Uveitis present	433	24.1	128	22.3	−1.8 (−6.2 to 2.6)
	Psoriasis present	203	11.3	59	10.3	−1.0 (−3.9 to 1.9)
	IBD present	190	10.6	54	9.4	−1.2 (−4.0 to 1.6)
	Dactylitis present	69	3.8	25	4.3	0.5 (−1.2 to 2.2)
	Enthesitis present	174	9.9	65	11.3	1.4 (−1.36 to 4.4)
	PJD present	320	17.8	116	20.2	2.4 (−1.7 to 6.5)
Patient-reported characteristics						
		**n**	**Mean (SD**)	**n**	**Mean (SD**)	**Mean difference (95% CI**)
Referral delay†	Years	1784	7.8 (9.8)	571	8.6 (10.2)	0.8 (−0.2 to 1.7)
Disease activity	BASDAI: 0 (best) – 10 (worst)	1364	4.9 (2.6)	454	4.7 (2.5)	−0.2 (−0.4 to 0.1)
Physical function	BASFI: 0 (best) – 10 (worst)	1374	4.6 (2.9)	461	4.7 (2.8)	0.1 (−0.4 to 0.2)
Spinal mobility	BASMI: 0 (best) – 10 (worst)	1288	3.9 (2.0)	453	3.9 (1.9)	0 (−02 to 0.3)
Global health	BASG: 0 (best) – 10 (worst)	1364	5.2 (2.8)	458	4.9 (2.7)	−0.3 (−0.5 to 0.1)
Spinal pain	VAS: 0 (best) – 10 (worst)	1366	4.5 (3.0)	457	4.4 (2.9)	−0.1 (−0.4 to 0.2)
SF-12 MCS*	Scored: 0 (worst) – 100 (best)	1332	45.3 (11.6)	455	47.6 (11.1)	2.3 (1.1 to 3.5)
SF-12 PCS	Scored: 0 (worst) – 100 (best)	1332	38.4 (11.9)	455	38.1 (11.9)	−0.3 (−1.6 to 1.0)
Quality of life	ASQoL: 0 (best) – 18 (worst)	1362	8.8 (5.8)	458	8.3 (5.5)	−0.5 (−1.1 to 0.1)
Anxiety*	HADS: 0 (best) – 21 (worst)	1362	7.9 (4.8)	455	7.1 (4.6)	−0.8 (−1.3 to −0.3)
Depression*	HADS: 0 (best) – 21 (worst)	1362	6.1 (4.2)	455	5.5 (4.1)	−0.6 (−1.1 to −0.2)
Sleep disturbance	Jenkins Sleep Evaluation Questionnaire: 0 (best) – 20 (worst)	1368	10.5 (6.2)	461	10.0 (6.4)	−0.5 (−1.2 to 0.1)
Fatigue*	CFS: 0 (best) – 11 (worst)	1379	4.3 (3.8)	463	3.9 (3.7)	−0.4 (−0.8 to −0.04)
Work absenteeism	%	760	6.8 (20.2)	258	5.8 (17.6)	−1.0 (−3.7 to 1.8)
Work presenteeism	%	758	31.2 (27.1)	258	31.8 (25.8)	0.6 (−3.2 to 4.4)
Overall work impairment	%	737	32.3 (28.0)	251	33.3 (27.2)	1.0 (−3.0 to 5.0)
Activity impairment	%	1350	44.4 (30.0)	452	42.5 (28.5)	−1.9 (−5.1 to 1.2)

*Indicates significant difference between urban and rural dwellers (p<0.05).

†Delay from symptom onset to first referral to specialist clinic.

ASQoL, Ankylosing Spondylitis Quality of Life Index; BASDAI, Bath Ankylosing Spondylitis Disease Activity Index; BASFI, Bath Ankylosing Spondylitis Functional Index; BASG, Bath Ankylosing Spondylitis Patient Global Score; BASMI, Bath Ankylosing Spondylitis Metrology Index; CFS, Chalder Fatigue Scale; DMARD, disease-modifying antirheumatic drugs; ESM, extraspinal manifestations; HADS, Hospital Anxiety and Depression Scale; IBD, inflammatory bowel disease; NSAID, non-steroidal anti-inflammatory drug; PJD, peripheral joint disease; SF-12 MCS, Short Form-12 Mental Component Score; SF-12 PCS, Short Form-12 Physical Component Score; VAS, Visual Analogue Scale.

Rural participants scored better than urban dwellers in terms of local area deprivation, mental health (SF-12 Mental Component Score, HADS anxiety, HADS depression) and fatigue. The groups were not significantly different in gender distribution, alcohol use, medication, prevalent extraspinal manifestations, disease activity, physical function, spinal mobility, quality of life or sleep disturbance.

**Figure 1:**
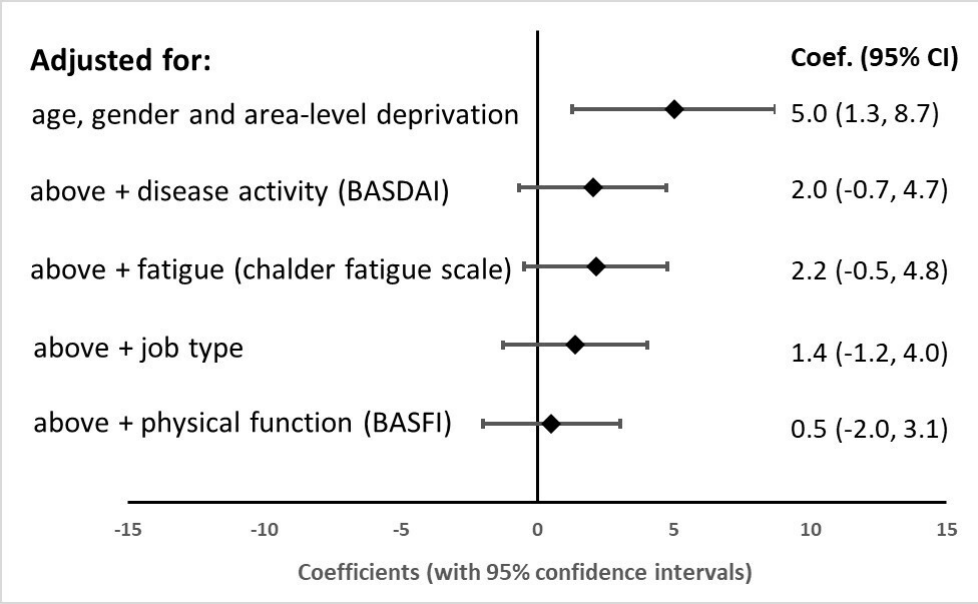
Relationship between presenteeism and living in a rural area, adjusted for increasing number of factors. BASDAI, Bath Ankylosing Spondylitis Disease Activity Index; BASFI, Bath Ankylosing Spondylitis Functional Index.

After adjusting for age, gender and local area deprivation, living in rural areas was associated with employment in a mainly physical/labour-intensive job (OR. 2.0 (95% CI 1.5 to 2.6)). Rural patients indicated that they experienced higher levels of work presenteeism and overall work impairment compared with urban patients (5.0% (1.3% to 8.7%) and 5.5% (1.6% to 9.4%)). After additional adjustment for disease activity (coefficient 2.0 (95% CI −0.7 to 4.7)), fatigue (2.2 (–0.5 to 4.8)), job type (1.4 (−1.2 to 4.0)) and physical function, the relationship between presenteeism and rural dwelling was attenuated (0.5 (−2.0 to 3.1)) (figure 1).

#### Association between living in a rural area and outcome of treatment with biologic therapy

Of the BSRBR-AS participants, 421 commenced biologic therapy and provided information at 12-month follow-up. Of these, 26% lived in a rural area. Similar differences were noted between the urban and rural participants commencing a biologic therapy, to those seen in the whole BSRBR-AS cohort. Biologic patients living in rural areas were older, exhibited lower levels of local area deprivation and a longer referral delay but lower anxiety scores (see online supplementary table S1). They were more likely to have been HLA-B27-typed and work in a physical job. The proportion of biologic patients in employment was similar between rural and urban dwellers (38.7% and 36.5%, respectively) and to that observed in the whole BSRBR-AS cohort. Similar rates of response in ASAS20 and ASAS40 criteria were seen among rural and urban patients at 12-month follow-up (OR of response in rural patients 0.9 (0.6 to 1.5) and 1.0 (0.6 to 1.6), respectively) (table 2).

**Table 2:** Associations with living in rural areas and outcome among those commencing a biologic therapy

	Univariable regression analyses, adjusted for age gender, local area deprivation and baseline score (as appropriate)		
Categorical outcomes		**OR**	**95% CI**
ASAS response criteria	ASAS20 met	0.93	(0.59 to 1.47)
	ASAS40 met	0.96	(0.58 to 1.57)
Continuous outcomes		**Coef**	**95% CI**
Disease activity	BASDAI: 0 (best) – 10 (worst)	−0.05	(−0.56 to 0.46)
Physical function	BASFI: 0 (best) – 10 (worst)	0.17	(−0.46 to 0.50)
Quality of life	ASQoL: 0 (best) – 18 (worst)	0.06	(−0.99 to 1.11)
Fatigue	CFS: 0 (best) – 11 (worst)	−0.16	(−0.94 to 0.62)
Sleep disturbance	Jenkins Sleep Evaluation Questionnaire: 0 (best) – 20 (worst)	0.32	(−0.80 to 1.44)
Anxiety	HADS: 0 (best) – 21 (worst)	0.22	(−0.50 to 0.95)
Depression	HADS: 0 (best) – 21 (worst)	−0.35	(−1.07 to 0.37)
Work absenteeism	%	1.52	(−3.78 to 6.81)
Work presenteeism*	%	9.96	(2.85 to 17.06)
Overall work impairment*	%	10.37	(2.63 to 18.10)
Activity impairment	%	0.74	(−4.84 to 6.32)

*Indicates significant difference between urban and rural dwellers (p<0.05).

ASAS, Assessment in Ankylosing Spondylitis; ASQoL, Ankylosing Spondylitis Quality of Life Index; BASDAI, Bath Ankylosing Spondylitis Disease Activity Index; BASFI, Bath Ankylosing Spondylitis Functional Index; CFS, Chalder Fatigue Scale; Coef, β coefficients; HADS, Hospital Anxiety and Depression Scale.

**Figure 2:**
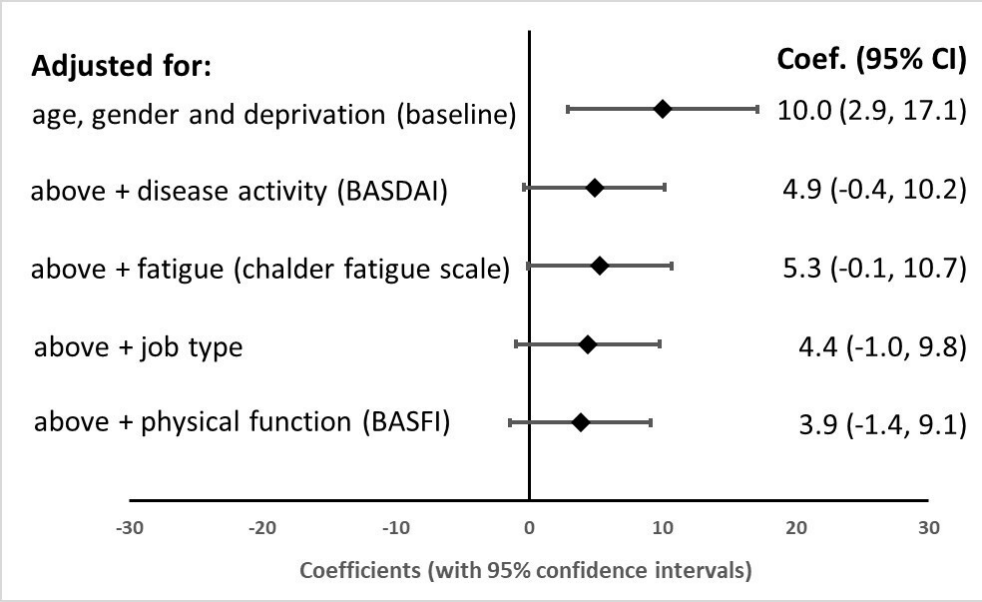
Relationship between presenteeism at follow-up and living in a rural area among biologic patients, adjusted for increasing number of factors. BASDAI, Bath Ankylosing Spondylitis Disease Activity Index; BASFI, Bath Ankylosing Spondylitis Functional Index.

Moreover, there were no significant differences in disease activity, physical function, quality of life, fatigue, sleep disturbance, anxiety, depression or activity impairment (outside work) at 12 months between rural and urban patients. However, among those currently in employment, living in a rural area was associated with a higher proportion of presenteeism and overall work impairment compared with urban dwellers (coefficient 10.0% (2.9% to 17.1%) and 10.4% (2.6% to 18.1%)). However, on additional adjustment for disease activity (coefficient 4.9% (95% CI −0.4% to 10.2%)), fatigue (5.3% (−0.1% to 10.7%)), job type (4.4% (−1.0 to 9.8%)) and physical function (3.9% (−1.4% to 9.1%)), the relationship between presenteeism and rural dwelling was also attenuated in the biologics cohort (figure 2).

### Interview findings

We conducted 30 semistructured telephone interviews with a subset of patients drawn from the BSRBR-AS registry; 77% were of working age across a broad spread of occupations, 80% were male, 60% had disease duration >11 years and 60% lived in a rural location (see online supplementary table S2).

#### People with axSpA want to work

Many interviewees reported benefits of being in work. In particular, work was important in terms of self-identity, providing social interactions as well as financial security.

…I enjoy the contact [with customers] and talking about the work, I do enjoy that really and how we’re going to solve a problem between us. Yeah, so I think I would really miss that actually. (40-002)

#### Ability to work negatively affected by axSpA

Nearly three-quarters of interviewees recognised that axSpA caused them challenges at work. In particular, reduced mobility, pain and fatigue impacted physical and mental function at work:

I’m a retail manager…there is a lot of physical work in it as well and sort of mental work with it and before the injections I did find it sort of like hard, I was restricted with what I could carry and what I can move about and things like that. (30-001)

It’s quite rough driving about in the machines so sometimes it’s really sore. (10-005)

The impacts were sufficiently important that they influenced decisions about type of work, choice of career and working hours:

I’ve always kind of done catering jobs, so I’ve been on my feet…but I don’t do that anymore in terms of obviously I can’t be on my feet for that length of time, and now I work in an office. (60-004)

I don’t think I would cope with full-time, just because I am very sore, I do get very tired. I think full-time is unrealistic for me at the minute, anyway. (60-001)

#### Job demand-control-support and flexibility at work

Job titles and simplistic classification of occupations, for example, sedentary/manual, failed to fully capture the physical demands of different types of work. No matter what the physical demands, however, people with axSpA who reported autonomy and control to perform their job or specific tasks how and when they wanted to were able to cope better at work. Employer flexibility and adjustments to working practices enabled participants to continue to work, when they would otherwise have been unable:

If I’m feeling poorly or whatever I phone my manager and we can…I’ll just do some work at home. (30-002)

They’ve been really good…they got me a more ergonomic chair and I’m allowed to get up if…when I need to, sort of every half an hour I’ll get up and have a little stretch and walk down the corridor and then come back to my desk. (60-004)

Support, from managers or colleagues, was another enabling factor:

If there’s something that needs doing fairly immediately and time is of the essence then someone will help me, or someone else has to do it and I’ll go and do something else. (20-001)

More challenges were experienced in workplaces where inflexibility was perceived:

The management within the company is very old-school and they’re not very flexible at all. They don’t promote working from home, they don’t like people coming in late, they don’t like people coming in early…the management have got clean bills of health, so they expect everyone else to. (10-001)

A number of participants reported that it was the lack of support and flexibility that had resulted in them leaving some jobs. Moreover, some types of work demands could be perceived as encouraging presenteeism even when participants were feeling unwell:

Part of the job was interviewing people…if you’re feeling unwell or not, you didn’t want to let people down by not turning up to interviews. I think that’s what drove it on. (30-003)

Contracts and entitlement to sick pay emerged as important factors influencing presenteeism. Some participants reflected that their experiences with Human Resources seemed punitive and disciplinary, rather than supportive, with a lack of understanding of the difference between minor illnesses and a long-term condition like axSpA:

There wasn’t any sort of relaxation, it was like, “Right, you’ve been off for so many days and these days, and you’re off again,” and they total the counts…it was unpleasant and to me, unnecessary…not a question of I’ve got a cold; I’ve got a major problem, you know? (30-003)

#### Disclosure to employers

Many participants had disclosed their condition to their employer because of the medication they were taking or because they needed time off to attend appointments, or their condition was physically obvious. However, employees reflected that there was reluctance on both sides to initiate discussion about the condition unless there were issues around work performance. Employers reportedly did not understand or care about the condition, and axSpA was often misunderstood as simply ‘a bad back’.

I don’t think they’d understand what it was anyway, it would just be words on a piece of paper…until it affected my time, my attendance, I don’t think they’d care. (50-005)

Others openly disclosed their condition, keen to ‘prove’ that employees with disabilities were a valuable asset:

That’s why I like telling people that I’ve got a condition because…my colleagues are surprised because you don’t see any difference in my performance to anybody else’s. (30-002)

#### Commuting to and from work

Despite sampling from rural and urban postcodes, most of the interviewees who lived in rural settings worked in urban settings. Adaptations to driving and commuting to work were commonplace:

I drive an automatic car now…I couldn’t manage without it…actually living here without a car in this particular location that we’re in, you would find it very difficult because we’re not on main bus routes…you need a car. (10-003)

The only trouble I do have is getting out, I find the car parking spaces a little bit too small if I can’t open the door wide to get out of the car. (60-003)

#### Effects of medication on work

Many participants reported that biologic drugs improved their ability to work. In contrast, use of opiate-containing analgesics caused problems, especially for those driving or operating machinery:

I cannot take my like heavy pain killers when I am operating my machinery. If it’s really really sore I have to put up with it or can’t go to work if it’s bad. (10-005)

#### Role of the rheumatology team

Participants rarely discussed work issues with their rheumatologist. Consultations were mainly focused around medical aspects of the disease and medications. Many felt that their healthcare professionals could be more proactive in discussing work issues, while a few reported that their care team provided support and encouragement to work:

They ask me every time I go. “Are you okay for work?”. [the Consultant] says…“it’s better for you if you can carry on because you’ve got something to look forward to and to get up and get yourself going.” So yes, they’re very supportive. (20-004)

## Discussion

Patients with axSpA living in rural areas were older, more likely to work in a physical job and reported more presenteeism and overall work impairment than urban dwellers. We found no overall differences in disease activity at baseline/follow-up, but adjustment at an individual level for clinical factors (disease activity, fatigue, physical function) and a crude measure of job type attenuated differences in work productivity. In the subset of patients with axSpA prescribed biologics, there were no differences in response to biologics, but after adjustment for age, sex and local area deprivation rural dwellers still reported more presenteeism and overall work impairment at follow-up. These work effects could similarly be explained by accounting for differences in disease activity, fatigue, physical function and job type.

Interviews with a subset of BSRBR-AS participants illustrated the complex relationship between clinical factors, contextual factors (such as working conditions, job demands) and work disability. The ability to work is of prime importance to patients and employment had health benefits. Flexibility in terms of what, when and how tasks are done is important. Job titles failed to reflect the heterogeneity of current working conditions: both physical and mental demands and the level of autonomy afforded to individuals to carry out their work as their health fluctuates. There was important variation in support offered by employers and how little people with axSpA perceive they can seek help with work from healthcare professionals. Together these findings suggest that work factors, health and healthcare are causing differential impacts on rural dwellers.

Our study has some limitations. We acknowledge that there are differences between rural and urban populations and have previously shown differences between working and non-working persons.^8^ However, in this study we have specifically focused on differences in working and non-working people in rural and urban areas. These were exploratory analyses, prioritised by patient representatives, which sought to investigate the full range of work outcomes, making no a priori assumptions. Future studies should focus on the effects of mediating factors and their role on the relationship between rurality and work outcomes, and will need to take account of the effect of multiple testing. However, irrespective of potential interactions between factors, poorer work outcomes for rural dwellers have important implications for patient care. We also recognise that presenteeism and work impairment data are skewed. Importantly, we have only used these data to assess descriptive differences between urban/rural patients and used linear regression to look at outcomes, and therefore this will not affect interpretation of our results.

Our study has a number of strengths. We employed nationally accepted definitions of rurality and have adjusted for local area deprivation. We have also measured presenteeism, which is increasingly recognised as commonly impacted by long-term conditions. However, productivity is difficult to define and existing self-reported measures of presenteeism (such as the Work Productivity and Activity Impairment, Health and Work Performance Questionnaire, Work Limitations Questionnaire, and Work Ability Index) have poor validity against objective markers and against each other^39 40^ and are oversimplistic in understanding work disability. Outcome Measures in Rheumatology Clinical Trials (OMERACT) colleagues^41^ have reached similar conclusions and stressed the relevance and importance of ‘contextual factors’ on impact from a health condition on work, for example, type of work, environment, and degree of flexibility. Our mixed-methods study adds quantitative and qualitative evidence about the relevance and nature of clinical and contextual factors, offering new insights into how features of occupations, individuals and workplaces influence work disability in axSpA. It illustrates the need for newer measures that do not take a self-assessed percentage loss approach but gauge impacts of health on work in a more comprehensive fashion.

In keeping with other studies, we found that patients with axSpA report significant levels of presenteeism,^7 8 42^ particularly in public-facing jobs (healthcare and hospitality), where there is a greater perception of ‘letting others down’.^43–45^ Others recognised that presenteeism was therapeutic, allowing them to maintain their social contact and sense of self-worth. Indeed, in a study of young adults with axSpA, they emphasised that work was a priority for them in order to establish a career, financially support young family and maintain identity.^46^

Our finding that rural dwellers have more issues with work productivity is novel but perhaps not surprising. Rural settings have both more restrictive labour markets alongside greater difficulties for individuals accessing local healthcare services.^9 13–16^ Our rural dwellers were older and more likely to be doing physically demanding occupations. This suggests that they have less flexibility to access work more suitable for their health. Many people living in rural settings were travelling to urban settings to work, suggesting another important issue for rural dwellers is the need to commute as well as undertake their work each day.

Emergent themes were coherent with the model of demand-control and support proposed by Karasek and Theorell.^47^ Karasek and Theorell 47postulated that people could tolerate very high levels of workplace mental and physical demands providing that they were offered autonomy and control over how to work and support from colleagues and managers to deliver their work. Among people with axSpA, we found flexibility about work tasks was a key enabler of ongoing work participation. Similarly, while not specifically focused on work, other qualitative studies in axSpA have highlighted the need for flexibility to adapt daily working practices in response to unpredictable flares, as well as influencing choice of career and decisions to change job.^46 48^ Career flexibility was important, especially in those with more physical jobs.^46^ While we found that work colleagues were a source of considerable support in enabling task flexibility, other studies have reported that unsatisfactory or slow work was a source of potential conflict with colleagues and employers.^49^ The differing ways in which people now participate with work also raise questions about how we define presenteeism, sick absence and fitness for work.

Our findings have important implications for practice, policy and research. Healthcare professionals could do much more to support their patients to work well, and resources are becoming available to support this.^50^ Most employers will have very few employees with rheumatic disease, and do not understand the often unpredictable disease course, and relapsing and remitting trajectory. Education and support for employers to enable flexibility at work could be transformative, particularly if ‘best practice’ examples from other employers were made available.

New work measures are needed to capture important contextual factors and comprehensively determine the impact of long-term conditions in the workplace, specifically taking account of the different ways in which people now engage in work. More broadly the role of geographical location in health and work outcomes in rheumatic disease is another important area for future research. The current European League Against Rheumatism axSpA recommendations (which emphasise that improving quality of life is the key aim) are focused only on disease activity and function.^51^ Future recommendations should include support to work well as a target for management to optimise quality of life in patients with axSpA.
