# Supplementary material for: Outcomes and treatment responses, including work productivity, among people with axial spondyloarthritis living in urban and rural areas: a mixed-methods study within a national register
Source: Ann Rheum Dis. 2020 Jun 10;79(8):1055–62. doi: 10.1136/annrheumdis-2020-216988 (PMC7392479; doi:10.1136/annrheumdis-2020-216988)
Supplement: Supplementary data [file annrheumdis-2020-216988supp001.pdf]

Supplementary Material

Table S1. Baseline characteristics of urban and rural dwellers commencing a biologic therapy

**S1**	**Baseline characteristics of urban and rural dwellers commencing a biologic therapy**					
		**Urban Dwellers**		**Rural Dwellers**		**Difference (rural vs. urban)**
Demographic characteristics						
		**N.**	**mean (SD)**	**N.**	**mean (SD)**	**mean diff (95% CI)**
**Age***	years	309	45.5 (13.3)	110	51.1 (13.2)	-5.6 (-8.5, -2.7)
**Deprivation***	1(most) – 5(least)	310	3.0 (1.5)	111	3.7 (1.1)	-0.6 (-0.9, -0.3)
		**N.**	**%**	**N.**	**%**	**mean % diff (95% CI)**
**Gender**	Male	213	68.7	74	66.7	-2.0 (-19.6, 15.6)
**Smoking Status**	Never	128	41.6	47	43.1	1.5 (-12.6, 15.6)
	Ex	102	33.1	42	38.5	5.4 (-7.7, 18.5)
	Current	78	25.3	20	18.4	-6.9 (-16.5, 2.7)
**Alcohol**	Never	29	9.4	7	6.4	-3.0 (-8.4, 2.4)
	Ex	54	17.5	22	20.2	2.7 (-6.7, 12.1)
	Current	225	73.1	80	73.4	0.3 (-18.3, 18.9)
**Employed**	Yes	195	63.5	68	61.3	2.2 (-19.2, 14.7)
**Job type***	Mainly desk/sedentary	103	54.8	22	33.3	-21.5 (-38.8, -4.2)
	Mainly physical/labour intense	85	45.2	44	66.7	21.5 (-0.2, 43.2)
Clinical characteristics						
**HLA B27 status***	Tested	219	70.6	85	76.6	6.0 (-12.7, 24.6)
**NSAID**	Prescribed (last 6m)	229	74.4	87	78.4	4.0 (-15.0, 23.0)
**DMARD**	Prescribed (last 6m)	33	15.3	14	18.2	2.9 (-7.7, 13.5)
**ESM (history)**	Uveitis present	82	26.6	22	19.8	-6.8 (-16.6, 3.0)
	Psoriasis present	38	12.3	10	9.0	-3.3 (-9.8, 3.2)
	IBD present	40	13.0	11	9.9	-3.1 (-9.9, 3.7)
	Dactylitis present	16	5.2	8	7.2	2.0 (-3.2, 7.2)
	Enthesitis present	41	13.3	13	11.7	-1.6 (-8.8, 5.6)
	PJD present	74	24.0	24	21.6	-2.4 (-12.4, 7.6)
Patient reported characteristics						
		**N.**	**mean (SD)**	**N.**	**mean (SD)**	**mean diff (95% CI)**
**Referral delay***	Years†	302	7.1 (9.3)	108	9.7 (11.0)	-2.6 (-4.7, -0.4)
**Disease Activity**	BASDAI: 0 (best) – 10 (worst)	307	6.4 (2.0)	109	6.4 (2.0)	0.05 (-0.4, 0.5)
**Physical Function**	BASFI: 0 (best) – 10 (worst)	309	6.2 (2.4)	111	6.3 (2.5)	-0.03 (-0.5, 0.5)
**Spinal Mobility**	BASMI: 0 (best) – 10 (worst)	235	4.3 (1.8)	87	4.5 (1.8)	-0.2 (-0.6, 0.3)

**Global Health**	BASG: 0 (best) – 10 (worst)	306	7.1 (1.8)	110	7.1 (1.7)	0.04 (-0.3, 0.4)
**Spinal Pain**	VAS: 0 (best) – 10 (worst)	308	6.3 (2.4)	110	6.3 (2.5)	0.03 (-0.5, 0.6)
**SF12 MCS**	Scored: 0 (worst) – 100 (best)	297	42.6 (11.5)	110	44.2 (11.4)	-1.6 (-4.1, 0.9)
**SF12 PCS**	Scored: 0 (worst) – 100 (best)	297	33.0 (10.4)	110	31.8 (10.1)	1.3 (-1.0, 3.5)
**Quality of Life**	ASQoL: 0 (best) – 18 (worst)	305	11.9 (4.6)	110	11.8 (4.4)	0.1 (-0.9, 1.1)
**Anxiety***	HADS: 0 (best) – 21 (worst)	307	9.3 (4.5)	109	8.2 (4.0)	1.1 (0.1, 2.1)
**Depression**	HADS: 0 (best) – 21 (worst)	307	7.5 (4.0)	109	7.4 (4.2)	0.7 (-0.8, 1.0)
**Sleep Disturbance**	Jenkins: 0 (best) – 20 (worst)	308	12.9 (5.7)	111	12.8 (6.1)	0.1 (-1.3, 1.3)
**Fatigue**	CFS: 0 (best) – 11 (worst)	310	5.6 (3.5)	111	5.5 (3.9)	0.2 (-0.6, 0.9)
**Work absenteeism**	%	174	10.1 (24.5)	64	13.7 (26.8)	-3.6 (10.8, 3.7)
**Work presenteeism**	%	169	44.6 (25.7)	63	44.6 (25.8)	-0.05 (-7.5, 7.4)
**Overall Work impair.**	%	165	45.8 (26.0)	61	47.7 (27.5)	-1.8 (-9.6, 6.0)
**Activity impairment**	%	303	60.0 (24.7)	110	58.8 (25.0)	1.2 (-4.2, 6.6)

* indicate significant difference between urban and rural dwellers (p<0.05)

† delay from symptom onset to first referral to specialist clinic

ACR – American college of rheumatology; ASQoL – Ankylosing Spondylitis Quality of Life Index; BASDAI – Bath Ankylosing Spondylitis Disease Activity Index; BASFI – Bath Ankylosing Spondylitis Functional Index; BAS-G - Bath Ankylosing Spondylitis Patient Global Score: BASMI - Bath Ankylosing Spondylitis Metrology Index; CFS – Chalder Fatigue Scale; ESM – extra spinal manifestations; HADS – Hospital Anxiety and Depression Scale; IBD – inflammatory bowel disease; PJD – peripheral joint disease; SF-12 MCS – Short Form 12 Mental Component Score; SF-12 PCS – Short Form 12 Physical Component Score; VAS – visual analogue scale.

Table S2. Interview participant characteristics

**S2**	**Interview participant characteristics**
**Characteristic**	**Number of participants**
**Age**	
18 – 44	10
45 – 64	13
≥ 65	7
**Gender**	
Female	6
Male	24
**Location**	
Rural	18
Urban	12
**Time since diagnosis**	
0 to 4 years	2
5 to 10 years	7
11 plus	18
Not specified	3
**ONS Standard Occupational Classification (SOC)**	
Administrative and secretarial	6
Associate professional and technical	2
Caring, leisure and other service occupations	2
Elementary e.g. agricultural, construction occupations	4
Managers, directors and senior officials	4
Process, plant and machine operatives	1
Professional occupations	5
Sales and customer services	2
Skilled trades	4

Supplementary Text: Interview topic guide

Opening question: ***"Can you talk me through your experiences of living with ankylosing spondylitis?"***

IMPACT OF ANKYLOSING SPONDYLITIS

1. Impact of disease on function and quality of life

1.1 *How long have you had Ankylosing Spondylitis?*

1.2 *How does Ankylosing Spondylitis affect you?*

1.3 *What are the main problems you have as a result of it?* **Probe**: Pain, stiffness, fatigue, sleep, reduced mobility, difficulty performing daily activities, and self-confidence.

2. Treatment

2.1 *What treatment are you currently receiving to manage your Ankylosing Spondylitis?*

2.2 *Is the treatment working for you at the moment?*

2.3 How easy is it for you to access healthcare services?

WORK & WORK ENVIRONMENT

3. Can you talk me through your work/employment history?

4. Current/past work (questions in past tense if not working/unemployed)

4.1 *Are you currently working (e.g part-time/full time)?*

<FOLLOW- If yes>

4.2 *What job do you do?*

4.3 *What does your job entail?*

4.4 *Can you describe to me a typical day at work?*

4.5 *Do you need to drive as part of your job? If so how often e.g. how many hours per week?*

5. Work setting/work environment

5.1 *How would you describe your work environment?*

Probe: large scale/small scale/ established organisational structure vs. small scale setting

5.2 *Roughly, how many people work for your employer/company (if not self-employed)?*

5.3 *What kind of occupational health support does your company provide?*

6. Commuting to work

6.1 *How would you describe your commute to work every day? How long does it normally take? **Follow on*** Do you drive? Can you park when you get there?

6.2 *Overall, how are you managing with your daily commute?*

WORK DISABILITY

7. Impact of disease on work performance

7.1 *Does Ankylosing Spondylitis affect you at work?*

<FOLLOW- If yes> *Are there any particular aspects of the job/task that you find difficult?*

What do you struggle the most with at work? **Probe**: pain/fatigue/cognitive symptoms

7.2 *Have you taken any days off work (sick leave) due to Ankylosing Spondylitis? If yes, how frequent? How many days over the past month?*

7.3 *Have you ever attended work when you have been unwell?*

<FOLLOW- If yes> Ask for examples Not just unwell but have got a flare up of AS?

7.4 How much flexibility do you think you have at work?

8. Relationship with employer

8.1 *Have you discussed your diagnosis with your employer?*

<FOLLOW- If yes> *If yes; what was their reaction to this? Was this helpful, if not why not?*

8.2 *Does your employer monitor absence?*

8.3 *Have you had any problems with your employer because of issues related to your Ankylosing Spondylitis e.g. time off work, being unable to do specific jobs/tasks?*

9. Adaptations to work

9.1 *Have you changed your job since your diagnosis?*

<FOLLOW- If yes> *Why?* **Probe:** To avoid certain strenuous tasks, working long hours or jobs that are not flexible and cannot be adjusted to their needs.

9.2 *Have you had to make any changes/adaptations to your work routine and tasks due to the condition?*

<FOLLOW- If yes> *What changes have you made?* **Probe**: reduced hours, full-time to part-time, and change in job roles/duties due to health condition.

9.3 *Do you think Ankylosing Spondylitis has affected your career progression/ opportunities?* **Probe**: early retirement, passed on promotional opportunity, accepting less demanding roles, and avoid strenuous tasks

10. Coping strategies

10.1 *How else do you manage your condition at work?*

<FOLLOW> *Can you give examples of the strategies you use?*

10.2 *What are the most effective strategies you use?*

<FOLLOW> *How do they help?*

SUPPORT

11. Provision of support by clinical team

11.1 *Have you talked to a healthcare professional about work worries?*

<FOLLOW- If no> *Why not?*

11.2 *Do you find it difficult to get to appointments about your AS or for blood tests?*

11.3 *Do you have to take annual leave for your appointments or can you go in paid work time? Could we make your appointments more convenient in any way?*

11.4 *Have you been offered any support in terms of work issues e.g. occupational advice? Been referred to an OT?*

11.5 *Is there occupational health at your workplace? Have they been involved?*

<FOLLOW> *Do you feel that you were supported adequately by the clinical team?*

12. Areas for improvement

12.1 *What kind of support would be useful to help you at work?*

<FOLLOW- If yes> *Who do you feel is best placed to provide this support?*

12.2 *Is there anything that you would specifically like more information/support with?*

12.2 *How would you like to receive support e.g. face to face meeting with person, via telephone, website, written information leaflets?*

13. Communications with work colleagues

13.1 *Are your work colleagues aware of your diagnosis of Ankylosing Spondylitis?*

<FOLLOW- If yes> *If yes; what was their reaction to this? Was it helpful for them to know? If not, why not?* **Probe:** Fear of being stigmatised, perceptions of vulnerability.

13.2 *Do you feel your colleagues understand you condition?*

13.3 *Has Ankylosing Spondylitis affected your relationship with your colleagues?*

IMPACT ON LIFE

14. Impact of work disability on social, family, and financial security

14.1 *Is it important for you to work?*

14.2 How have work problems (e.g. time off work due to ill-health, unemployment) affected other aspects of your life?

Probe: *family life, social life, finances?*

15. Fears and concerns relating to work

15.1 *Do you have concerns about your ability to work, now and in the future?*

Probe: concerns - financial security, job security, employability, reduced opportunities, and unpredictability of condition.

<FOLLOW- If yes> *Has this affected your mood, for example resulted in anxiety or depression?*

Closing questions:

Is there anything else you would like to tell me?

Are there any questions you thought we would ask that we haven't asked?
